# A Case of Compression of the Brain, Reported to the Medical Society of Tennessee, at Its 13th Annual Meeting in May, 1842

**Published:** 1842-11

**Authors:** John R. Wilson

**Affiliations:** Davidson County


### WESTERN JOURNAL

OF

### MEDICINE AND SURGERY.

---

NOVEMBER, 1842.

---

ART. I.—*A Case of Compression of the Brain, reported to the Medical Society of Tennessee, at its 13th annual meeting in May*, 1842. By JOHN R. WILSON, M. D., of Davidson County.

Nancy, 32 years of age, the servant of Wm. H. Robertson, Esq., of Rutherford County, during the seventh month of her sixth pregnancy, was attacked with severe pain in the left side of the head, involving the temple, eye, &c., and with pain in the lumbar region, but not severe; her bowels were costive, tongue furred, pulse full and strong, appetite capricious. I bled her freely from the arm, administered a dose of castor oil, had a cold wet towel applied to her head, which had more than ordinary degree of heat. I visited her next day and found all the symptoms somewhat relieved; ordered light diet, and a repetition of the oil when necessary to prevent constipation. This course enabled her to pass her time with only an occasional recurrence of pain in the left side of her head, to which she said she had been subject many years, until about three weeks after the birth of her child, at which time I was called to see her again and found her suffering from pain as in the first attack, with but little arterial excitement, surface cold and clammy, tongue much furred, left eye slightly inflamed, no appetite, tinnitus aurium, and bowels confined. An emetic of ipecac. was administered at noon, 15 grs. blue pill ordered at bed-time, to be followed by a dose of oil next morning. During the next morning I visited her again; the oil had operated, but every symptom was worse—a hard tumor, circumscribed and slightly elevated, was discovered on the left side of the os frontis; near the junction of the parietal bone; there was strong full pulse, hot skin, occasional aberrations of mind, intolerance of light and jactitation. I bled her copiously, but without any relief of pain; gave 20 grs. calomel, shaved the tumor, and ordered oil in six hours, with warm fomentations to the tumor and temple. I visited her next day and found all the symptoms better except the pain in the tumor, in which she complained of severe pain and throbbing. I applied a blister plaster over the tumor and administered a dose of senna and salts. Next day the blister had drawn well, and the tumor being now soft, I opened it by a large incision, when it discharged a considerable quantity of purulent matter. An emollient poultice was applied, and directions were given to repeat it, with a view to keeping the orifice open.

For two days after the opening of this tumor the patient appeared relieved. On the third day she became extremely restless and unable to sleep, with want of feeling in the right hand and arm, which gradually extended to the lower extremity of the same side. During the ensuing night complete paralysis of the right side took place, attended with laborious breathing and stupor. The patient returned no answer that could be understood to any question asked; pulse slow and oppressed, but regular. A free crucial incision was made through the tumor, and on examination a portion of the pericranium was separated from the skull, and a small hole was discovered sufficient to admit the end of a probe through the first table of the bone, but no farther. The trephine was applied immediately, and as soon as a portion of the bone had been removed, four or five ounces of purulent matter were discharged. This was in a short time followed by a return of the power of distinct articulation and of a correct sense of hearing, and the patient was soon able to answer any question asked. The bone had lost its natural appearance as far as the dura mater was detached, which was to some extent, and several crowns of the trephine were taken out, so as to remove as far as possible all the diseased bone, which resembled wood of very hard texture irregularly worm-eaten. The dura mater had a dusky red appearance. Simple dressings of the ordinary kind, with gentle laxatives and light diet, soon enabled the patient to recover her health, which has continued without much interruption up to the present time—a period of more than 14 years. I would further remark that the patient had not received any mechanical injury on her head, but was of a family disposed to scrofulous diathesis, some of her brothers having since died of that disease.

May, 1842.

